# Supplementary material for: Estimating the potential impact and diagnostic requirements for SARS-CoV-2 test-and-treat programs
Source: Nat Commun. 2023 Dec 2;14:7981. doi: 10.1038/s41467-023-43769-z (PMC10693634; doi:10.1038/s41467-023-43769-z)
Supplement: Supplementary file 3 — Reporting Summary [file 41467_2023_43769_MOESM3_ESM.pdf]

nature portfolio

Corresponding author(s): Colin A. Russell
Last updated by author(s): Nov 12, 2023

Reporting Summary

Nature Portfolio wishes to improve the reproducibility of the work that we publish. This form provides structure for consistency and transparency in reporting. For further information on Nature Portfolio policies, see our Editorial Policies and the Editorial Policy Checklist.

Statistics

For all statistical analyses, confirm that the following items are present in the figure legend, table legend, main text, or Methods section.

n/a	Confirmed	
☐	☒	The exact sample size (*n*) for each experimental group/condition, given as a discrete number and unit of measurement
☒	☐	A statement on whether measurements were taken from distinct samples or whether the same sample was measured repeatedly
☒	☐	The statistical test(s) used AND whether they are one- or two-sided<br>*Only common tests should be described solely by name; describe more complex techniques in the Methods section.*
☒	☐	A description of all covariates tested
☐	☒	A description of any assumptions or corrections, such as tests of normality and adjustment for multiple comparisons
☐	☒	A full description of the statistical parameters including central tendency (e.g. means) or other basic estimates (e.g. regression coefficient) AND variation (e.g. standard deviation) or associated estimates of uncertainty (e.g. confidence intervals)
☒	☐	For null hypothesis testing, the test statistic (e.g. *F*, *t*, *r*) with confidence intervals, effect sizes, degrees of freedom and *P* value noted<br>*Give P values as exact values whenever suitable.*
☒	☐	For Bayesian analysis, information on the choice of priors and Markov chain Monte Carlo settings
☒	☐	For hierarchical and complex designs, identification of the appropriate level for tests and full reporting of outcomes
☒	☐	Estimates of effect sizes (e.g. Cohen's *d*, Pearson's *r*), indicating how they were calculated

Our web collection on statistics for biologists contains articles on many of the points above.

Software and code

Policy information about availability of computer code

Data collection	No software was used for data collection.
Data analysis	The PATAT model source code and custom code used to analyzed our simulation data are available at https://github.com/AMC-LAEB/PATAT-sim and https://github.com/AMC-LAEB/PATAT-sim/blob/main/projects/av_therapeutics/han-et-al_av_therapeutics.ipynb respectively.

For manuscripts utilizing custom algorithms or software that are central to the research but not yet described in published literature, software must be made available to editors and reviewers. We strongly encourage code deposition in a community repository (e.g. GitHub). See the Nature Portfolio guidelines for submitting code & software for further information.

Data

Policy information about availability of data

All manuscripts must include a data availability statement. This statement should provide the following information, where applicable:
- Accession codes, unique identifiers, or web links for publicly available datasets
- A description of any restrictions on data availability
- For clinical datasets or third party data, please ensure that the statement adheres to our policy

Data on global testing rates were downloaded from https://www.finddx.org/covid-19/test-tracker. All data relevant to the study are included in the Article, the Supplementary Information and the GitHub repository (https://github.com/AMC-LAEB/PATAT-sim).

Research involving human participants, their data, or biological material

Policy information about studies with human participants or human data. See also policy information about sex, gender (identity/presentation), and sexual orientation and race, ethnicity and racism.

Reporting on sex and gender	This study does not involve any human research participants.
Reporting on race, ethnicity, or other socially relevant groupings	This study does not involve any human research participants.
Population characteristics	This study does not involve any human research participants.
Recruitment	This study does not involve any human research participants.
Ethics oversight	This study does not involve any human research participants.

Note that full information on the approval of the study protocol must also be provided in the manuscript.

Field-specific reporting

Please select the one below that is the best fit for your research. If you are not sure, read the appropriate sections before making your selection.

☒ Life sciences ☐ Behavioural & social sciences ☐ Ecological, evolutionary & environmental sciences

For a reference copy of the document with all sections, see nature.com/documents/nr-reporting-summary-flat.pdf

Life sciences study design

All studies must disclose on these points even when the disclosure is negative.

Sample size	No sample size calculation was performed. This is an agent-based modeling study where we simulated SARS-CoV-2 epidemics in a population of 1,000,000 individuals to study how clinical testing rates affect the population-level impact of mass distribution of oral antivirals. This population size was chosen as it is sufficiently large enough to generate the desired epidemic characteristics using reasonable amount of computing resources and computation time. We validated our simulation results based on this population size against estimated SARS-CoV-2 prevalence in the UK in 2023 (see Model Validation in Supplementary Information).
Data exclusions	N/A. Only simulation data generated from our agent-based model is used in this study and none of them was excluded from our study.
Replication	We performed five independent, successful epidemic simulations for each unique parameter set.
Randomization	N/A. This is a descriptive study.
Blinding	N/A. This is a descriptive study.

Reporting for specific materials, systems and methods

We require information from authors about some types of materials, experimental systems and methods used in many studies. Here, indicate whether each material, system or method listed is relevant to your study. If you are not sure if a list item applies to your research, read the appropriate section before selecting a response.

Materials & experimental systems

n/a	Involved in the study	
☒	☐	Antibodies
☒	☐	Eukaryotic cell lines
☒	☐	Palaeontology and archaeology
☒	☐	Animals and other organisms
☒	☐	Clinical data
☒	☐	Dual use research of concern
☒	☐	Plants

Methods

n/a	Involved in the study	
☒	☐	ChIP-seq
☒	☐	Flow cytometry
☒	☐	MRI-based neuroimaging
